# A co-culture assay of embryonic zebrafish hearts to assess migration of epicardial cells *in vitro*

**DOI:** 10.1186/s12861-015-0100-y

**Published:** 2015-12-29

**Authors:** Monica S. Yue, Jessica S. Plavicki, Xin-yi Li, Richard E. Peterson, Warren Heideman

**Affiliations:** Molecular and Environmental Toxicology Center, University of Wisconsin, 1300 University Avenue, Madison, WI 53706 USA; Pharmaceutical Sciences Division, School of Pharmacy, University of Wisconsin, 777 Highland Avenue, Madison, WI 53705 USA; College of Life Science, Shaanxi Normal University, Xi’an, Shaanxi 710062 China

**Keywords:** Epicardial cell, Epicardium, Proepicardium, Myocardial cell, Myocardium, Migration, Co-culture

## Abstract

**Background:**

The vertebrate heart consists of three cell layers: the innermost endothelium, the contractile myocardium and the outermost epicardium. The epicardium is vital for heart development and function, and forms from epicardial progenitor cells (EPCs), which migrate to the myocardium during early development. Disruptions in EPC migration and epicardium formation result in a number of cardiac malformations, many of which resemble congenital heart diseases in humans. Hence, it is important to understand the mechanisms that influence EPC migration and spreading in the developing heart. *In vitro* approaches heretofore have been limited to monolayer epicardial cell cultures, which may not fully capture the complex interactions that can occur between epicardial and myocardial cells *in vivo*.

**Results:**

Here we describe a novel *in vitro* co-culture assay for assessing epicardial cell migration using embryonic zebrafish hearts. We isolated donor hearts from embryonic zebrafish carrying an epicardial-specific fluorescent reporter after epicardial cells were present on the heart. These were co-cultured with recipient hearts expressing a myocardial-specific fluorescent reporter, isolated prior to EPC migration. Using this method, we can clearly visualize the movement of epicardial cells from the donor heart onto the myocardium of the recipient heart. We demonstrate the utility of this method by showing that epicardial cell migration is significantly delayed or absent when myocardial cells lack contractility and when myocardial cells are deficient in *tbx5* expression.

**Conclusions:**

We present a method to assess the migration of epicardial cells in an *in vitro* assay, wherein the migration of epicardial cells from a donor heart onto the myocardium of a recipient heart in co-culture is monitored and scored. The donor and recipient hearts can be independently manipulated, using either genetic tools or pharmacological agents. This allows flexibility in experimental design for determining the role that target genes/signaling pathways in specific cell types may have on epicardial cell migration.

**Electronic supplementary material:**

The online version of this article (doi:10.1186/s12861-015-0100-y) contains supplementary material, which is available to authorized users.

## Background

The heart is one of the first organs to form in vertebrate embryogenesis and during early development consists of three major cell layers: endocardium, myocardium, and epicardium. The endocardial and myocardial cells originate from populations of mesodermal cells that migrate from the midbrain-hindbrain boundary to form the linear heart tube [1, 2]. These cardiogenic mesoderm cells form the ventricle, atrium, outflow tract myocardium, and contribute to the cardiac conduction system [3]. The epicardium originates from a different population of cells, the proepicardium (PE), a transitory structure of progenitor cells arising from coelomic mesenchyme of the septum transversum [3]. Epicardial progenitor cells (EPCs) from the PE migrate onto the bare myocardium and envelop the heart, forming the epicardium. There are two known mechanisms of cell migration from the PE to the heart: 1) the release of free-floating EPC aggregates that land on the myocardium (e.g., mouse); 2) the formation of a tissue bridge between the PE and myocardium (e.g., chick). Both mechanisms of PE cell migration are observed in some species (e.g., zebrafish, axolotl) [4–6]. As the heart develops, a subset of epicardial cells undergo epithelial-to-mesenchymal transition and invade the subepicardial space. These mesenchymal cells, called epicardium-derived cells (EPDCs), are important for normal heart maturation and have been shown to differentiate into interstitial cardiac fibroblasts, coronary vascular smooth muscle cells, and adventitial fibroblasts. Though somewhat controversial, it is suggested that EPDCs also contribute to the coronary endothelium, valve development, myocardial cells, and Purkinje fiber differentiation [4, 7].

Congenital heart disease, which affects between 0.4-5 % of live births, is often due to defective cardiac morphogenesis involving problems with cardiac progenitor cells [3]. There has been increasing interest on epicardium formation and importance of the epicardium in subsequent heart development [7]. Ablation of the PE inhibits epicardium formation, causing an array of cardiac malformations that resemble malformations observed in human congenital heart disease. For example, chicks lacking an epicardium developed thin compact myocardia similar to human left ventricular non-compaction cardiomyopathy [8]. Aberrant crosstalk between the epicardial layer and underlying myocardial and endocardial cells has been implicated in several congenital diseases, such as hypoplastic left heart syndrome and endocardial fibroelastosis [3, 7].

*In vivo* approaches to studying PE migration and epicardium formation often include microsurgery to ablate the PE or the use of physical barriers to block migration. Manipulation of specific genes involved in signaling or cell adhesion has also been used to assess involvement in PE formation [5, 8–10]. However, the genes of interest, including *Wt1*, *Tbx18*, *Tcf21*, are expressed during development in other organs besides the heart: the use of mutants or morpholino oligonucleotide (MO) knockdown produces effects wherever the target gene is normally expressed. This creates a concern that the results have been influenced by altered gene expression not specific to heart cells [7, 9, 11].

Common *in vitro* approaches involve excising the PE or heart segment and monitoring effects on EPC migration in culture [9, 12, 13]. One advantage of this approach is the ease with which signaling factors can be added to the culture media to assess effects on migration [9]. In addition, it avoids the problem of off-target effects in gene manipulation experiments. However, most of these studies have focused on the effects of factors in the culture medium, rather than on the cell-cell interactions between epicardial cells and myocardial cells, which have been shown to play an important role in heart development *in vivo* [6, 8, 14].

Here we describe an *in vitro* assay to assess and quantify the migration of epicardial cells from a donor heart onto the bare myocardium of a recipient heart. In this assay, the important cell-cell interactions between different cell types remain intact. Because the technique cultures multiple cell types, differentiated cell phenotypes are better preserved [15], allowing the hearts to remain healthy in culture for several days. This permits for lengthy observations, not possible with most *in vitro* approaches. In this method, the source of migrating epicardial cells is different from the source of target myocardial cells, making it possible to manipulate either or both types of cells independently.

We use embryonic zebrafish as the source of hearts. This is advantageous for several reasons: zebrafish produce large numbers of offspring, embryonic hearts can be efficiently isolated, externally fertilized eggs allow for gene manipulation with MO or CRISPR-Cas9, and there is clear observation of effects during early development. Additionally, a variety of transgenic lines are readily available [1].

In this report, we demonstrate key features of this assay by assessing the ability of epicardial cells from a donor heart (marked with *tcf21*:DsRed2) to migrate onto the myocardial surface of a recipient heart (marked with *cmlc2*:EGFP). We show the normal course of migration, and how migration was inhibited when the recipient hearts were extracted from embryos injected with MOs against *silent heart* (*sih*), and *tbx5. Sih* morphants lack a heartbeat [16]. *Tbx5*, which is expressed in multiple tissues in the heart, has been implicated in EPC migration *in vivo* [12]. Because this approach maintains the multiple cell types of the *in vivo* setting, yet allows for manipulation of individual cell types, this assay can be used to identify not only genes important in epicardial formation, but also where they function.

## Methods

### Zebrafish

**Fig. 1:**
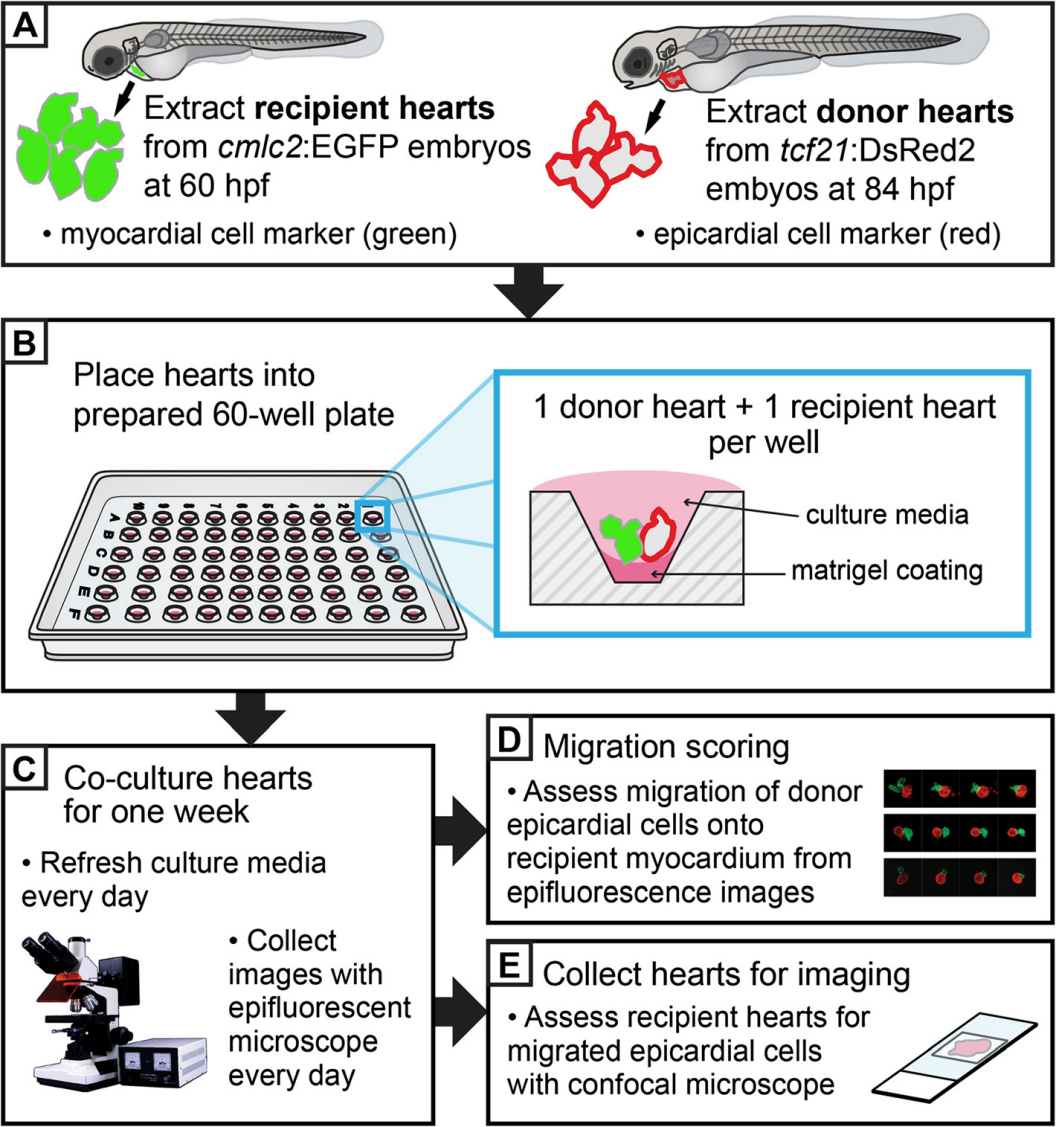
Overview schematic of the *in vitro* co-culture assay for assessing migration of epicardial cells. (**a**) Hearts are extracted from embryonic zebrafish. Recipient hearts are collected from *cmlc2*:EGFP embryos, which have a green myocardial cell marker. These hearts are extracted at 60 hpf, prior to the migration of epicardial progenitor cells (EPCs), such that extracted hearts have bare myocardia. Donor hearts are collected from *tcf21*:DsRed2 embryos, which have a red epicardial cell marker. These hearts are extracted at 84 hpf, after migration of EPCs has begun, such that extracted hearts carry some epicardial cells that are actively spreading. Recipient and/or donor embryos may be pre-treated before isolation of hearts according to experimental design. For example, in this report the recipient hearts came from embryos that were injected with MOs affecting expression of specific genes. (**b**) Collected hearts are placed in a prepared 60-well cell culture dish. Each well has been pre-coated with a thin layer of matrigel basement membrane mix, and contains one donor and one recipient heart, submerged in culture medium. The donor and recipient hearts are arranged such that the ventricles are in contact. (**c**) Donor and recipient hearts are co-cultured for one week. Each day the culture media is refreshed and each culture well is imaged with an epifluorescence microscope. (**d**) Epifluorescence images are scored in a blinded fashion for the migration of donor epicardial cells onto recipient myocardial cells. (**e**) After 7 days in culture, heart samples are fixed and stained for immunohistochemistry. Each sample is analyzed with confocal microscopy for presence of donor epicardial cells that have migrated onto recipient myocardia to verify positive migration

Embryos were obtained from adult zebrafish (*Danio rerio*) housed and maintained according to guidelines described in Westerfield (2000) [17]. Embryos were harvested at 84 h post fertilization (hpf) for obtaining “donor hearts”. These hearts were obtained from the transgenic line *tcf21*:DsRed2 [*Tg(tcf21:DsRed2)*^*pd37*^], which marks epicardial cells with a red fluorescent protein. The “recipient hearts” were collected from 60 hpf embryos from the transgenic line *cmlc2*:EGFP [*Tg(cmlc2:EGFP)*^*f1*^], which marks myocardial cells with a green fluorescent protein (Fig. 1a).

The *silent heart* (*sih*; cardiac troponin T2, *tnnt2*) and *tbx5* (T-box 5) MOs were obtained from Gene Tools (Philomath, OR). The Gene Tools standard control morpholino (control MO) was used as a control. The MO sequences were: *sih*, 5’ CATGTTTGCTCTGATCTGACACGA 3’ [16]; *tbx5*, 5’ GAAAGGTGTCTTCACTGTCCGCCAT 3’ [18]; control MO, 5’ CCTCTTACCTCAGTTACAATTTATA 3’. A 2 nM MO solution was prepared with either the *sih*, *tbx5*, or control MO, and microinjected into *cmlc2*:EGFP eggs in the 1-2 cell stage. Microinjections were done as previously described [19]. Eggs were collected into a petri dish with autoclave-sterilized egg water (60 μg/ml Instant Ocean Sea Salts with 0.2 ppm methylene blue). Embryos were screened for MO incorporation at 48 hpf, and only MO positive embryos were used. Clean water changes were made daily.

All procedures involving zebrafish were approved by the Animal Care and Use Committee of the University of Wisconsin-Madison and adhered to the National Institute of Health’s “Guide for the Care and Use of Laboratory Animals.”

### Culture media and plate preparation

Culture medium consisted of Leibovitz’s L-15 medium (Life Technologies) supplemented with 10 % fetal bovine serum (Life Technologies) and 4x penicillin/streptomycin (Fisher Scientific). The culture medium was filtered through a disposable sterile filter unit, and stored at 4 °C prior to use. Conical-bottom 60-well plates with lids (Electron Microscopy Sciences) were pre-plated with a mix made from Matrigel basement membrane matrix mix (Corning), with a protein concentration diluted to 4 mg/mL in 1X DMEM (Corning) and supplemented with 4x penicillin/streptomycin. The matrigel mix was prepared ahead of time in a sterile hood and stored at -20 °C. On the culture start day, the matrigel mix was thawed and plated onto a culture dish following the manufacturer-recommended thin gel coating method. Each well required approximately 10 μL of matrigel mix to coat. After the culture dish was set (37 °C, 30 min) it was stored in the cell culture incubator (28 °C, 5 % CO_2_) until time for placement of hearts into culture.

### Isolation of embryonic hearts and placement into culture

Hearts were extracted from embryos according to methods adapted from Burns and MacRae [20]. Briefly, 50 to 80 embryos were lightly anesthetized with tricaine (MS 222, Sigma-Aldrich) and placed into a 1.7 mL microcentrifuge tube. Excess water was drawn off and 1 mL of culture medium was added. The microcentrifuge tube with embryos was placed beneath a 5 mL syringe fitted with a 19-gauge needle, adjusted such that the beveled end of the needle aligned with the 0.25 mL mark on the microcentrifuge tube. The syringe was gently pumped up and down, bringing culture media and embryos into and out of the syringe, in a rhythm guided by beats on a metronome. Hydrodynamic shear forces remove the hearts from the bodies. These forces are proportional to the rate of flow through the needle, and inversely proportional to the needle diameter, thus the rate of syringe pumping is critical. All contents from the syringe and microcentrifuge tube were then quantitatively emptied from the syringe with washes and filtered through a 105 micron nylon mesh (Component Supply) to separate the bodies and other large debris from the hearts in the filtrate. If necessary, the media was filtered again with a 37 micron nylon mesh (Component Supply) to retain the hearts on the filter and remove smaller debris. Hearts in medium were placed in a Petri dish and collected using a micropipettor with the aid of an Olympus SZX16 epifluorescence stereomicroscope and the EGFP or RFP heart markers, and placed temporarily into a droplet of fresh culture media until all hearts had been collected. The efficiency of heart extraction yields varied depending on the number of strokes (total number of draws and expulsions) and rate of plunger motion; these factors differed depending on the age and treatment of the embryos. Donor hearts from 84 hpf *tcf21*:DsRed2 embryos required approximately 70 strokes (35 draws and 35 expulsions) at a rate of 60 beats per minute (bpm) according to a metronome. Recipient hearts from 60 hpf control MO *cmlc2*:EGFP embryos required approximately 50 strokes at a rate of 60 bpm. Recipient hearts from 60 hpf embryos injected with *sih* MO or *tbx5* MO were considerably more vulnerable to over-shearing that can destroy the tissue, and as such required between 40 to 50 strokes at 40 bpm for extraction.

Once all donor (*tcf21*:DsRed2) and recipient (control, *sih*, or *tbx5* MO *cmlc2*:EGFP) hearts were collected, 5 μL of fresh culture media was added to each well of the prepared culture dish. One donor heart and one recipient heart were added to each well with a minimal carry over of extra medium using a micropipettor. Sterilized forceps were used to gently arrange the hearts so that they lay side-by-side, with the ventricles in contact. This work was done under a dissecting microscope. After placement of hearts into culture was completed the culture dish was gently returned to the incubator, avoiding disturbances that might separate donors from recipients.

### Culture conditions

Culture medium was refreshed on a daily basis by removing up to 5 μL of old medium and adding 5 to 8 μL of fresh culture medium (Fig. 1c). The culture dish was carefully monitored for signs of contamination and any questionable samples were removed and not used for analysis. As previously mentioned, the cell culture incubator was maintained at 28 °C with 5 % CO_2_.

### Assay imaging and scoring

Beginning on the day after hearts were placed in culture, images of each well were obtained daily (Day 1 through Day 7 in culture) using an Olympus DP72 camera mounted on an Olympus SZX16 epifluorescence stereo microscope with cellSens software (Fig. 1c). Images were processed using Adobe Photoshop (Adobe). The migration of epicardial cells (red) from the donor heart onto the myocardium of a recipient heart (green) was scored by an experimenter blinded to sample identity (Fig. 1d). Scoring was based on a scale from 0 to 7: 0 = no migration of epicardial cells was observed during the duration of culture; 1 = epicardial cells were only observed on the recipient myocardium on Day 7; 2 = migration of epicardial cells began on Day 6 and continued to expand coverage of the recipient myocardium through Day 7; 3 = migration began on Day 5, etc.; 4 = migration began on Day 4, etc.; 5 = migration began on Day 3, etc.; 6 = migration began on Day 2, etc.; 7 = migration began on Day 1, etc.

### Assay analysis

Each culture dish well containing one donor and one recipient heart that remained in contact throughout the seven days in culture was considered *n* = 1 for statistics. In order to assess whether data for each treatment group could be pooled from two experimental replicates, two-way analysis of variance was conducted to confirm that variation from different experimental days did not have an effect. Since both the experimental day and interaction variables were not significant (*p* < 0.05) for both *sih* MO and *tbx5* MO groups and their respective controls, replicate data sets were pooled. The pooled sample size for the control vs. *sih* MO group was *n* = 13 to 14, and the pooled sample size for control vs. *tbx5* MO group was *n* = 14 to 19. Student’s *t-*test was used to compare the pooled migration scores of control MO recipient hearts with respective *sih* MO or *tbx5* MO recipient hearts. *F*-test was used to check homoscedasticity of data and significance was set at *p* < 0.05. All statistical analysis was conducted with GraphPad Prism statistics software (GraphPad Software).

### Immunohistochemistry

On Day 7, the donor/recipient hearts from each well were collected from the culture dish and processed for confocal imaging using a method from Plavicki et al. [6] (Fig. 1e). Primary antibody rabbit anti-DsRed2 (AnaSpec) was used in a 1:200 dilution in PBT (0.3 % Triton X-100 in phosphate buffered saline) buffer. Secondary antibody, anti-rabbit Alexa 568 antibody (Invitrogen), was used in a 1:100 dilution in PBT buffer. Confocal images were collected with an Olympus Fluoview FV1000 microscope. Brightest point projections were made using Olympus Fluoview software (Olympus) and images were processed in Adobe Photoshop (Adobe).

## Results

To verify that migration from a donor heart to a recipient heart can be assessed *in vitro*, we collected normal donor hearts from *tcf21*:DsRed2 embryos and placed them in culture with control MO recipient hearts from *cmlc2*:EGFP embryos. In zebrafish, EPCs begin migrating to the ventricle between 60-72 hpf. By 96 hpf, epicardial cells cover most of the ventricle, and, by 120 hpf, also cover most of the atrium. Hence, donor hearts were collected at 84 hpf, a time point at which epicardial cells were present and actively spreading on the ventricle. In contrast, recipient hearts were collected at 60 hpf before EPCs began migrating to the ventricle in order to prevent recipient-epicardial cells from confounding our results. To confirm that recipient hearts extracted at 60 hpf lacked epicardial cells, we examined hearts from embryos with both a red epicardial marker, *tcf21*:DsRed2, and green myocardial marker, *cmlc2*:EGFP. Hearts extracted from *cmlc2*:EGFP; *tcf21*:DsRed2 embryos at 60 hpf lacked epicardial cells on the ventricle and atrium (*n* = 10). Neither *tcf21*+ cells nor DAPI-stained cells with the flattened epicardial cell phenotype were observed on the myocardia of these hearts (Additional file 1: Figure S1 A). In addition, we confirmed that recipient hearts did not contain cells that were capable of independently differentiating into epicardial cells after 7 days in the presented culture conditions. Hearts from 60 hpf *cmlc2*:EGFP; *tcf21*:DsRed2 embryos that were individually maintained in culture for 7 days did not have any *tcf21*+ cells or DAPI-stained cells with the epicardial phenotype present on their myocardia (*n* = 8, Additional file 1: Figure S1 B).

**Fig. 2:**
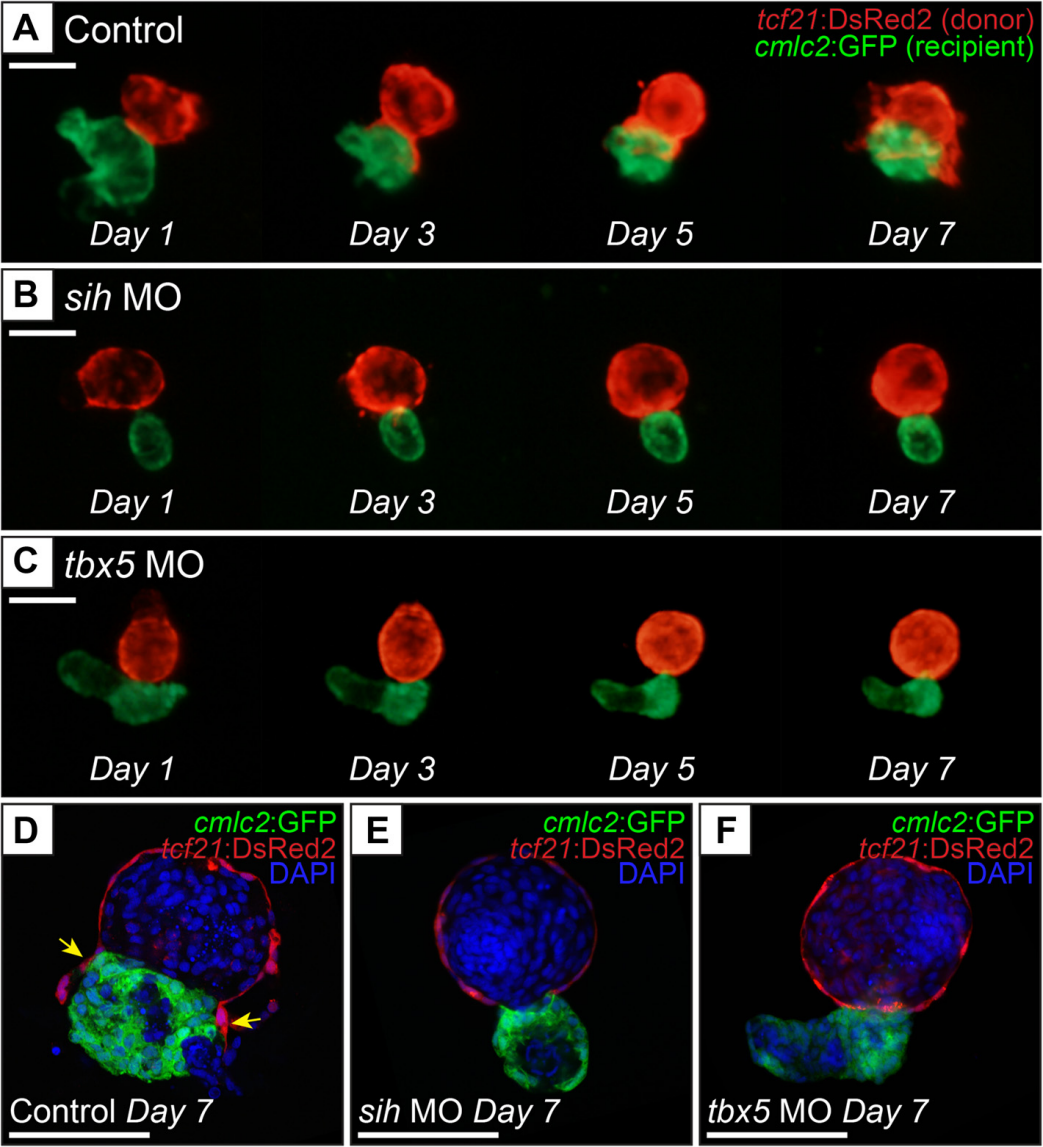
Migration of epicardial cells from donor hearts onto control, *sih*, or *tbx5* MO recipient hearts. **a**-**c** Fluorescence images taken on Days 1, 3, 5, and 7 in culture show progression of epicardial cell migration. Red (*tcf21*:DsRed2) shows epicardial cells from the donor, green (*cmlc2*:EGFP) shows recipient myocardial cells. **a** Migration of donor epicardial cells onto a control MO recipient heart is apparent by Day 5 in culture. The merged red-on-green signal, appearing yellow, is significantly noticeable by Day 7. There does not appear to be any significant migration of donor epicardial cells onto either the *sih* MO recipient heart (**b**) or the *tbx5* MO heart (**c**) throughout the 7 days in culture. **d**-**f** Confocal microscopy images of donor/recipient heart samples after 7 days in culture. Red indicates *tcf21*:DsRed2 donor epicardial cells, green indicates *cmlc2*:EGFP recipient myocardial cells, blue indicates DNA (DAPI stain). **d** Confocal microscopy verifies the presence of donor epicardial cells that have migrated onto the control MO recipient myocardium (yellow arrows). In contrast, there does not seem be any donor epicardial cells on the *sih* MO recipient heart (**e**) or the *tbx5* MO recipient heart (**f**), which is consistent with the epifluorescence images. In this figure, single hearts were followed throughout the 7-day time course in panels **a**-**c**, and were then collected to produce the confocal images shown in panels **d**-**f**. Scale bars in all images represent 100 μm

**Fig. 3:**
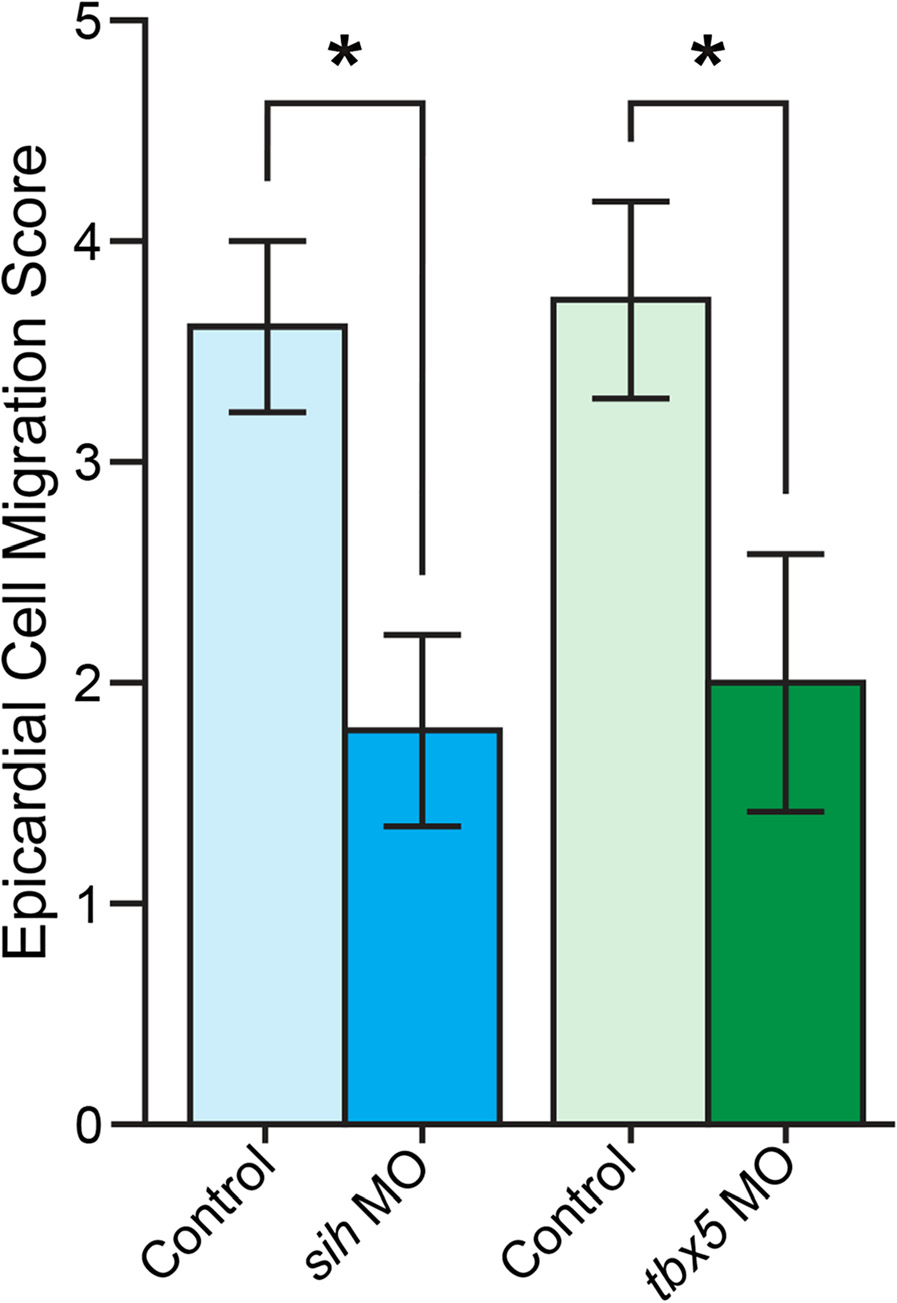
Migration scores for *sih* and *tbx5* MO recipient hearts are significantly lower than respective controls. Each sample was scored for migration of epicardial cells as described in the Methods. Bar graphs show average migration score for each group, error bars represent standard error of the mean (SEM), asterisk indicates that the treatment group is significantly different from its respective control (Student’s *t*-test, *p* < 0.05)

Scoring for epicardial cell migration was assessed by the increasing overlap between red epicardial signal (*tcf21*+) and green myocardial signal (*cmlc2*+) over time (Fig. 2a-c). In control experiments, epicardial cell migration was observed in 12 of 13 samples (controls for *sih* MO cohort) and 17 of 19 samples (controls for *tbx5* MO cohort). In general, epicardial cells from control samples showed clear signs of migration onto recipient myocardia between Day 4 and 5 (Figs. 2a, and 3). This was reflected in the scoring: average migration scores for control samples were 3.615 (SEM ±0.385) for the *sih* MO cohort, and 3.737 (SEM ±0.445) for the *tbx5* MO cohort (Fig. 3). In contrast, no migration was observed at all in 5 of 14 samples in the *sih* MO group. If migration occurred it was minimal and significantly delayed, beginning in most cases between Day 6 and 7 (Fig. 2b). This was reflected in a significantly lower average migration score of 1.786 (SEM ±0.435) (Fig. 3). Similarly, there was no migration in 6 of 14 samples in the *tbx5* MO group. Again, in those cases in which migration occurred the area of overlap was small and migration was significantly delayed, beginning in most cases on Day 6, with an average migration score of 2.000 (SEM ±0.584) (Fig. 3).

While migration can be readily observed with normal fluorescence microscopy, it can be difficult to determine whether the merged signal is due to true overlap of spreading epicardial cells in contact with the underlying myocardia; a merged signal can also result when the two tissues are simply positioned above and below each other but not in actual contact. Thus, migration was confirmed using confocal microscopy to examine samples on Day 7 for the presence of *tcf21*+ epicardial cells on top of, and associated closely with, the myocardial cells of recipient hearts (Fig. 2d-f). Donor epicardial cells (red) were observed covering recipient myocardial cells (green) in control samples (yellow arrows, Fig. 2d). In contrast, many recipient hearts from the *sih* MO and *tbx5* MO groups did not have any observable donor epicardial cells covering the labeled myocardial cells (Fig. 2e-f).

## Discussion

The heartbeat is halted in *sih* morphants [16]. Our results show that epicardial cell migration is significantly delayed or inhibited when the myocardial cells do not contract. This supports our previous results, which showed that pharmacological inhibition of heartbeat inhibited migration of PE cells and spreading of epicardial cells over the myocardium, both *in vitro* and *in vivo* [6]. A similar co-culture assay comparing migration of epicardial cells onto normal and *sih* MO recipient hearts was also presented in those experiments [6]. We have developed the assay further to increase precision: instead of mixing a large number of donor and recipient hearts in a 24-well culture dish, we have refined the method into using only one donor and one recipient heart per well in a culture dish. This reduces the number of hearts needed per experiment, and reduces the risk of widespread contamination across many hearts. This also addresses potential concerns that neighboring hearts could influence migration, for example, by locally increasing the concentration of a secreted paracrine factor.

Although *tbx5* is expressed in several cardiac tissues, many studies have focused on investigating the role of *tbx5* expression in the PE [12]. In zebrafish, *tbx5a* is required for PE specification, a process that also involves BMP (bone morphogenetic protein) signals [21]. In the chick, *tbx5* expression is regulated in concert with initiation and cessation of cell migration. Either reducing or increasing *tbx5* expression in PE explants could inhibit EPC migration, *in vivo* and *in vitro* [12]. The investigators assessed *in vitro* migration as the ability of an epicardial monolayer of cells to spread out (migrate) in a cell culture dish. However, this approach does not provide for the crosstalk that may occur with myocardial cells, which also express *tbx5* [12]. Here, we show that inhibiting expression of *tbx5* in the myocardium alone is sufficient to substantially affect epicardial cell migration. Our results add to our overall understanding of *tbx5* in epicardium development.

It is important to recognize that in the presented assay the age of the recipient myocardial cells is different than that of the migrating donor epicardial cells, an interaction that does not happen in natural circumstances. It was necessary to use younger recipient myocardial cells in this assay so that recipient hearts lacked epicardial cells, which could influence the migration of donor epicardial cells and confound the interpretation of results. In chick models, it is possible to remove the source of epicardial cells by blocking or ablating the PE using microsurgery techniques [8, 10]. However this is difficult to replicate in zebrafish larvae. The size of the zebrafish PE is considerably smaller, making physical manipulations logistically challenging. Furthermore, multiple PEs form over multiple days of development and contribute to the zebrafish epicardium [6]. Therefore, a single ablation event cannot remove the PE.

Another possible approach is to genetically ablate epicardial cells from the recipient heart *in vivo* before extraction and placement into culture with a same-age donor heart. For example, bacterial nitroreductase can be expressed in epicardial tissues using the *tcf21* promoter to convert nontoxic metronidazole into a cytotoxin in *tcf21*+ cells [22]. However, complete ablation of epicardial cells is difficult, especially given the regenerative capacity of the zebrafish heart, as surviving epicardial cells are capable of repopulating the epicardium [22, 23]. Given these challenges, we felt that using the 60 hpf recipient heart was appropriate for the intended scope of this assay.

It is desirable to use genetic tools and techniques to study developmental processes such as epicardium formation *in vivo,* however, these genetic approaches rely on the availability of a cell-specific marker to drive expression of a recombinase, transcriptional factor, or other activating enzyme in a discrete expression pattern [24, 25]. While there are well-documented examples of myocardial-specific markers (e.g., *cmlc2*), there are no known PE- or EPC-specific markers that are not expressed in other tissues during development. Most common markers of the epicardial lineage, *Wt1*, *Tbx18*, *Tcf21*, are expressed in other tissues, including the liver, kidney, pectoral fin mesenchyme, developing palate, and pharyngeal arches [26–29]. Furthermore, the PE and epicardium are composed of heterogeneous populations of cells [30], for example, there are both *tcf21*+ and *tcf21*- cells in the zebrafish epicardium [6]. Therefore, a truly precise genetic approach would require targeted modifications using intersectional epicardial markers (e.g., use a dual recombinase approach to target gene expression in cells that are both *tcf21*+ and *tbx18*+). Designing and establishing such highly specific transgenic lines would take considerable time and effort. Hence, *in vitro* approaches, such as the one presented here, are desirable as comparatively faster and less logistically complex alternatives. The presented assay can aide in identifying candidate genes involved in EPC migration and provide insight into the tissue-specific role of these genes, while using readily available genetic tools.

## Conclusions

In conclusion, we have developed an assay that can assess epicardial cell migration *in vitro* by co-culture of a donor and recipient heart. Our assay uses whole hearts in culture, allowing for important cell-to-cell interactions between the epicardial and myocardial cells. Given that donor and recipient hearts come from different individuals, these cells can be uniquely manipulated in order to determine how effects in each cell type can influence EPC/epicardial cell migration. In addition to genetic manipulation, signaling factors, blocking antibodies, or pharmacologic agents can be readily added to the hearts before or after placement in culture media. Using our assay, we demonstrated that epicardial cell migration was inhibited when myocardial cells lacked contractility (*sih* MO). In addition, we demonstrated that lack of *tbx5* expression in myocardial cells alone was sufficient to inhibit epicardial cell migration.

## Additional file

Additional file 1: Figure S1. Hearts extracted at 60 hpf lack epicardial cells. (A and B) Confocal micrographs of *cmlc2*:EGFP; *tcf21*:DsRed2 hearts extracted at 60 hpf . Images show brightest point projections from confocal z-series. (A) *cmlc2*:EGFP; *tcf21*:DsRed2 hearts before being placed into culture (Day 0). (B), *cmlc2*:EGFP; *tcf21*:DsRed2 hearts after 7 days in culture (Day 7). There were no epicardial cells (red) observed on the heart myocardia (green) at Day 0 or Day 7. In addition, there were no observed *tcf21*- cells with the stereotypical flattened phenotype of epicardial cells present on top of the myocardium (blue, DAPI nuclear staining). Scale bars in all images represent 50 μm. (PNG 965 kb)

## Abbreviations

bpm: Beats per minute
BMP: Bone morphogenetic protein
*cmlc2*: Cardiac myosin light chain 2
DAPI: 4’,6-diamidino-2-phenylindole
DMEM: Dulbecco’s modified Eagle’s medium
DsRed2: variant of *Discosoma* sp. red fluorescent protein
EGFP: Enhanced green fluorescent protein
EPC: Epicardial progenitor cell
EPDC: Epicardium-derived cell
hpf: Hours post fertilization
MO: Morpholino oligonucleotide
PBT: 0.3 % Triton X-100 in phosphate buffered saline
PE: Proepicardium
SEM: Standard error of the mean
*sih*: Silent heart
*Tbx18*: T-box 18
*tbx5*: T-box 5
*tcf21*: Transcription factor 21
*tnnt2*: Cardiac troponin T2
*Wt1*: Wilms tumor 1
