# Supplementary figures and images for: A co-culture assay of embryonic zebrafish hearts to assess migration of epicardial cells in vitro
Source: BMC Dev Biol. 2015 Dec 29;15:50. doi: 10.1186/s12861-015-0100-y (PMC4696273; doi:10.1186/s12861-015-0100-y)

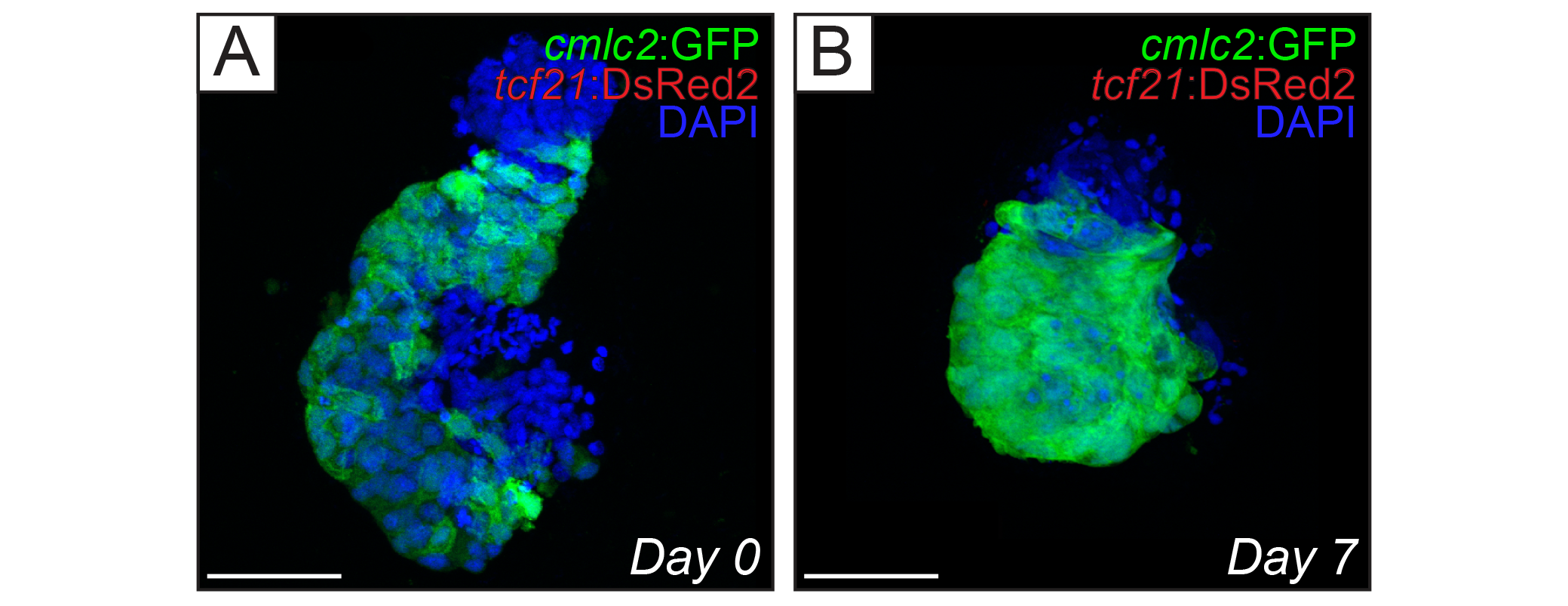

Supplement: Additional file 1: Figure S1. — Hearts extracted at 60 hpf lack epicardial cells. (A and B) Confocal micrographs of cmlc2:EGFP; tcf21:DsRed2 hearts extracted at 60 hpf . Images show brightest point projections from confocal z-series. (A) cmlc2:EGFP; tcf21:DsRed2 hearts before being placed into culture (Day 0). (B), cmlc2:EGFP; tcf21:DsRed2 hearts after 7 days in culture (Day 7). There were no epicardial cells (red) observed on the heart myocardia (green) at Day 0 or Day 7. In addition, there were no observed tcf21- cells with the stereotypical flattened phenotype of epicardial cells present on top of the myocardium (blue, DAPI nuclear staining). Scale bars in all images represent 50 μm. (PNG 965 kb) [file 12861_2015_100_MOESM1_ESM.png]
